# A clinical trial on the consistency of bilateral testicular tissue histopathology and Johnsen score: single side or bilateral side biopsy?

**DOI:** 10.18632/oncotarget.24748

**Published:** 2018-05-08

**Authors:** Wen-Hao Tang, Shan-Jie Zhou, Shi-De Song, Hui-Ying He, Han Wu, Zhe Zhang, Yu-Zhuo Yang, Hong-Liang Zhang, Jia-Ming Mao, De-Feng Liu, Lian-Ming Zhao, Hao-Cheng Lin, Kai Hong, Lu-Lin Ma, Xin-Jie Zhuang, Hui Jiang

**Affiliations:** ^1^ Department of Urology, Peking University Third Hospital, Beijing 100191, China; ^2^ Reproductive Medicine Centre, Peking University International Hospital, Beijing 102206, China; ^3^ Department of Urology, Rizhao People’s Hospital, Rizhao, Shandong 276826, China; ^4^ Department of Pathology, Peking University, Health Science Center, Beijing 100191, China; ^5^ Center for Reproductive Medicine, Department of Obstetrics and Gynecology, Peking University Third Hospital, Beijing 100191, China

**Keywords:** azoospermia, nonobstructive, male infertility, biopsy, pathology

## Abstract

To evaluate and compare left and right testicular tissue histopathology and Johnsen score, and to investigate the necessity for bilateral testicular biopsy. We recruited180 patients with non-obstructiveazoospermia (NOA) on testicular biopsy who had undergonetesticular sperm aspiration (TESA). Pathological sections of testicular tissue were diagnosed by specially-assigned doctors, who evaluated pathological findings, determined the Johnsen score and confirmed for the presence or absence of sperm. Sperm positive rates for left and right testicular histopathology were 55.0% and 51.7% respectively, and the proportion of Johnsen scores≥8 for left and right testes were 53.3% and 50.0%, respectively. Cohen kappa values revealed that the identification of sperm in bilateral testicular samples was not consistent and was related to random effects; Optimized cut-off value for bilateral testicular volume was 11ml (Johnsen score ≥8), and optimized cut-off values of E_2_ on left and right testes were 144.5pmol/L and 133.5 pmol/L (Johnsen score≤7). However, age, serum prolactin (PRL), follicle stimulating hormone (FSH), luteinizing hormone (LH) and total testosterone (TT) levels were not accurate predictors for the existence of testicular sperm. There was nostatistical significance between left and right testicular histopathology in terms of sperm positive rates or Johnsen score; the Johnsen score were caused entirely by random effects and a score from one side could not represent the other side. Therefore, we recommend that both testes need to undergo surgery when NOA patients undergo testicular biopsy or sperm retrieval.

## INTRODUCTION

Owing to developments in andrology, reproductive medicine and assisted reproductive technology (ART), it has been possible to use testicular sperm retrieval to allow patients with non-obstructive azoospermia (NOA) to father their own children. It is important to differentiate patients with NOA from those with obstructive azoospermia (OA), and exclude azoospermia caused by endocrine and genetic etiologies. In order to achieve successful diagnosis and provide appropriate therapy for NOA patients, it is necessary for andrologists to carry out testicular biopsy and testicular sperm aspiration/extraction (TESA/TESE), or select one of these two techniques to confirm the existence or absence of testicular sperm and evaluate the positive rate of sperm retrieval before commencing a cycle of intracytoplasmic sperm injection (ICSI). The European Association of Urology (EAU) guidelines (2010) recommend that a testicular biopsy is the best procedure for histological diagnosis and offers the best possibility of finding sperm in men with NOA [1]. In addition, several studies have concluded that testicular biopsy was the best predictor of a successful TESA/TESE, and that the strongest predictor of the success of TESA/TESE was when tubules were found in histopathology specimens which contained mature spermatozoa (Johnsen score ≥8). The lower limit threshold of 2% of tubules showing active spermatogenesis in a histopathology specimen would result in a positive sperm retrieval and retrieval rate of 50% for NOA patients [2]. However, sperm retrieval rates (SSR) for NOA patients vary according to the surgical procedure used. From 1112 NOA patients undergoing TESE, Tang et al. successfully found sperm in 44.78% (cell suspension examination) and 46.22% (histopathology) of NOA testicular tissue samples. Moreover, the consistency rate for these two detection methods was 92.63% with an overall sperm detection rate of 41.82%[3]. If using the microdissection testicular sperm extraction (micro-TESE) technique, the positive rate for sperm retrieval has been reported as45%–63% or 50% of the NOA males investigated [4]. Another research study claimed that the performance of micro-TESE was 1.5 times more likely (95% confidence intervals [CI]: 1.4–1.6) to result in successful sperm retrieval as compared to conventional TESE (cTESE), and that the performance of cTESE was 2.0 times more likely (95% CI: 1.8–2.2) to result in successful sperm retrieval as compared with TESA [5, 6]. In general, the SRR of cTESE is lower than micro-TESE, and consequently, physicians have agreed that micro-TESE should be considered as the gold standard for the retrieval of testicular sperm in NOA patients, and that a significant proportion of men undergoing micro-TESE would have successful sperm retrieval irrespective of previous histology or previous unsuccessful surgery [7].

The majority of researchers have agreed that testicular histology significantly influences SRR for NOA patients [8]. For example, testicular sperm were retrieved in 44.3% of patients but SRR was significantly higher in patients with hypospermatogenesis compared to patients with sertoli cell only syndrome (SCOS) or maturation arrest [7, 9, 10]. The histopathological examination of testicular tissue can qualitatively and quantitatively evaluate the status of spermatogenesis and the severity of dysfunction. Johnsen scoring is a commonly-used quantitative method and is used to evaluate and analyze testicular histology on a scale from 1 to 10, with a higher score representing a better status of spermatogenesis and a lower score representing more severe dysfunction. Sperm could still be identified in testicular tissue even when the Johnsen score ≥8. Moreover, following intensive research in spermatogenesis, and improvements to the testicular sperm retrieval procedure, an increasing number of NOA patients have been able to father their own children. The most commonly-used surgical procedures include TESA, TESE, testicular fine needle aspiration (TFNA) and micro-TESE, with the latter having the highest rate of sperm retrieval.

It is essential to establish the status of spermatogenesis and forecast the necessity and possible success rate of sperm retrieval procedures such as testicular biopsy, either in advance of the procedure or at the same time as sperm retrieval surgery. There is a general consensus of opinion amongst physicians that the spermatogenic status of bilateral testes is equal and that histopathology findings of a unilateral testis sample could represent both testes, at least if the volume and texture of the testes were the same or similar bilaterally. Otherwise, biopsy can be carried out on a pre-selected testicle unilaterally, for example, if one testis is diagnosed as having a larger volume and better texture than the other. However, different hospitals tend to adopt their own therapeutic procedure and sperm cryopreservation technique; consequently, the outcome from testicular biopsy is likely to differ across different hospital sites.

In this present study, 180 NOA patients who were undergoing testicular biopsy and investigated Johnsen score and bilateral testicular histopathology to assess the success rate of sperm retrieval, whether unilateral biopsy was representative of testicular status bilaterally, and whether it was necessary to carry out bilateral surgeries.

## RESULTS

### General patient data

**Figure 1:**
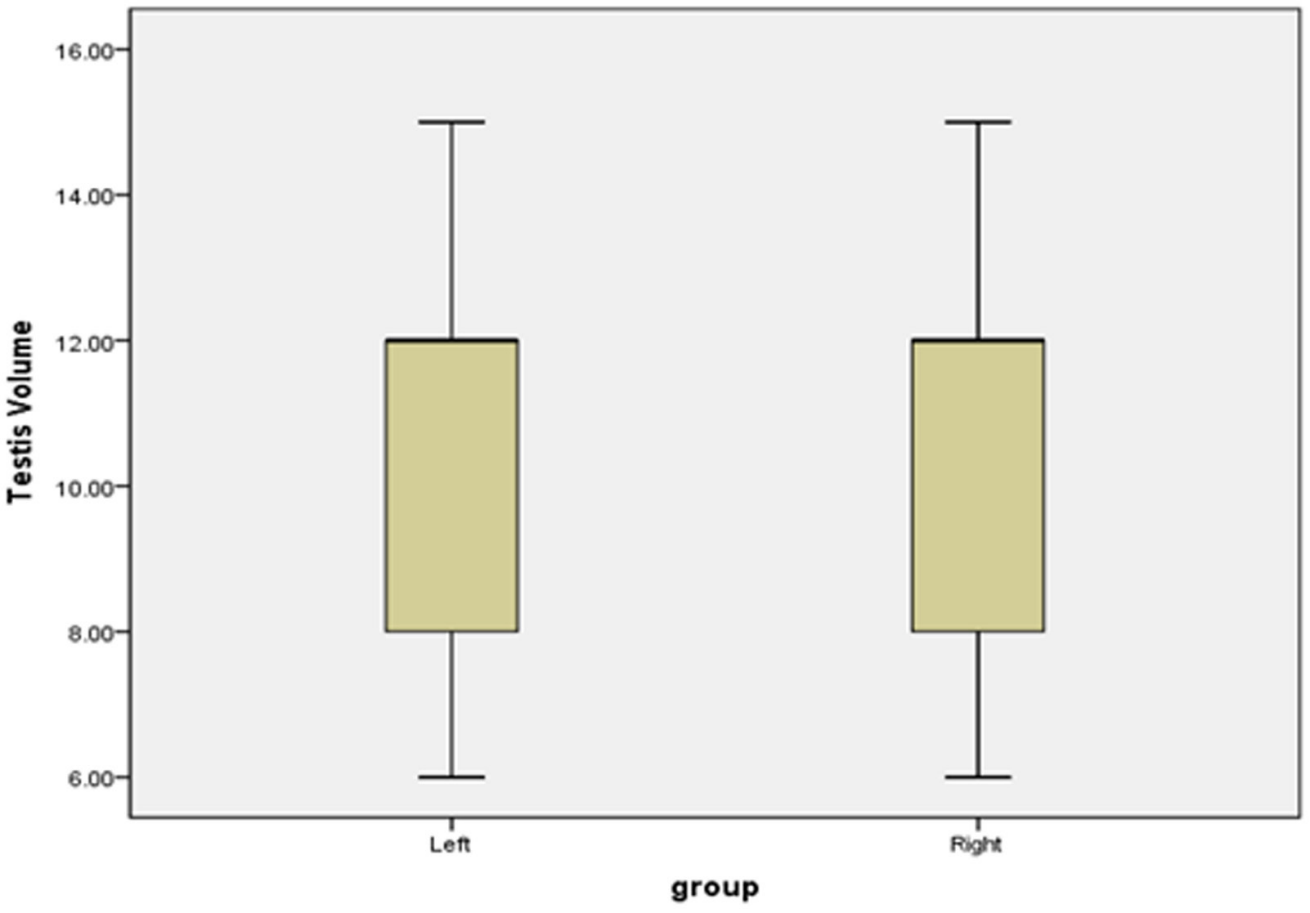
Boxplots of bilateral testicular volume

**Figure 2:**
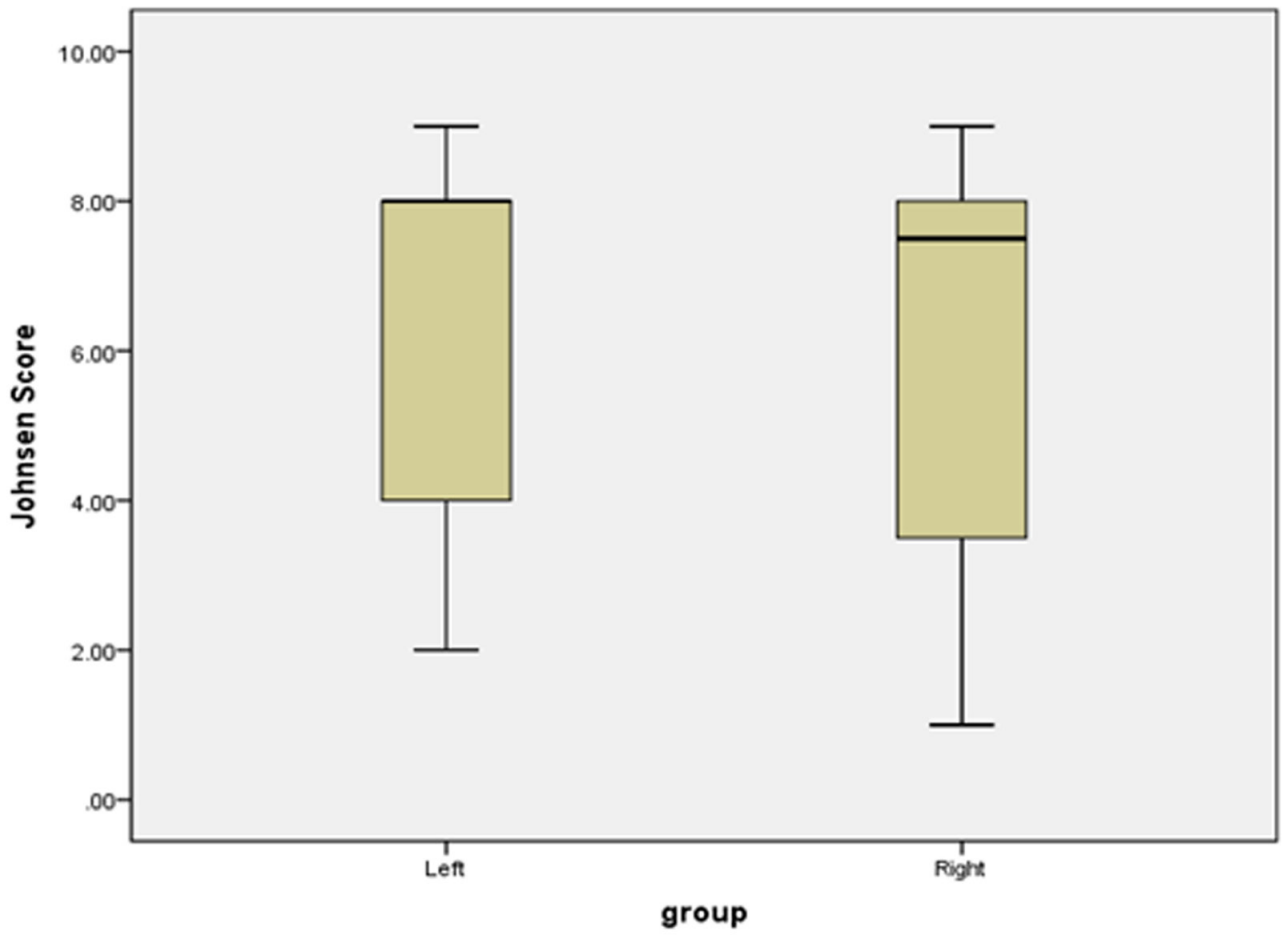
Boxplots of bilateral testicular Johnsen score

General data from the 180 patients are presented in Table 1; bilateral testicular volume was unequal in 25.0% of these patients. There was a statistically significant difference between the left and right testicular volume (*p*=0.022), but not between left and right Johnsen score (*p*=0.458). Box plots for testicular volume and Johnsen score are presented in Figures 1 and 2.

**Table 1:** Frequencies of 180 patients’ testicular volume, Johnsen score, age and serum reproductive hormone levels

	Mean	Standard deviation	Minimum value	Maximum value	*p* value (compare between left and right)
Left testicular volume (ml)	10.50	2.99	6.0	15.0	
Right testicular volume (ml)	10.05	2.79	6.0	15.0	0.022^*^
Johnsen score of left testis	6.20	2.58	2.0	9.0	
Johnsen score of right testis	6.07	2.73	1.0	9.0	0.458^#^
Age (years-old)	31.40	5.63	20.0	42.0	
PRL (ng/ml)	14.53	8.23	1.07	41.70	
FSH (mIU/ml)	4.83	2.14	1.36	14.99	
LH (mIU/ml)	3.65	1.69	1.17	11.17	
TT (nmol/L)	11.31	4.08	4.09	29.10	
E_2_ (pmol/L)	122.04	50.94	9.50	236.00	

^*^ Left and right testes: Paired-Samples T Test.

^#^ Left and right testicular Johnsen score: Two-Related-Samples Test (Wilcoxon Signed Ranks Test).

### Comparison of sperm positive rate and histopathology between the left and right testes

Sperm positive rates for left and right testicular histopathology were 55.0% and 51.7% respectively. Pearson’sχ^2^ test revealed there was no statistically significant difference between these rates (*p*=0.526). In terms of finding sperm in the testes bilaterally, we determined that the Cohen kappa value was 0.011(*p*=0.526); this related to mild consistency intensity and thus suggested that the consistency of finding sperm in the bilateral testes was caused by random effects, and was not consistent (Table 2).

**Table 2:** Finding sperm positive rates and Johnsen score of bilateral testicular

Group (testicular volume)	Finding sperm positive rates				Johnsen score ≥8			
	Left (%)	Right (%)	χ^2^ *p* value	Kappa value	Left (%)	Right (%)	χ^2^ *p* value	Kappa value
Total	55.0	51.7	0.526	0.011	53.3	50.0	0.266	0.000
6-8ml subgroup	18.2	15.4	0.654	0.008	18.2	19.2	0.278	−0.029
10-12ml subgroup	73.3	75.9	0.699	0.010	70.0	75.9	0.011	0.010
>12ml subgroup	87.5	100	0.154	0.068	87.5	60.0	P=0.006	0.000

### Comparison of johnsen scores for left and right testicular tissue

**Figure 3:**
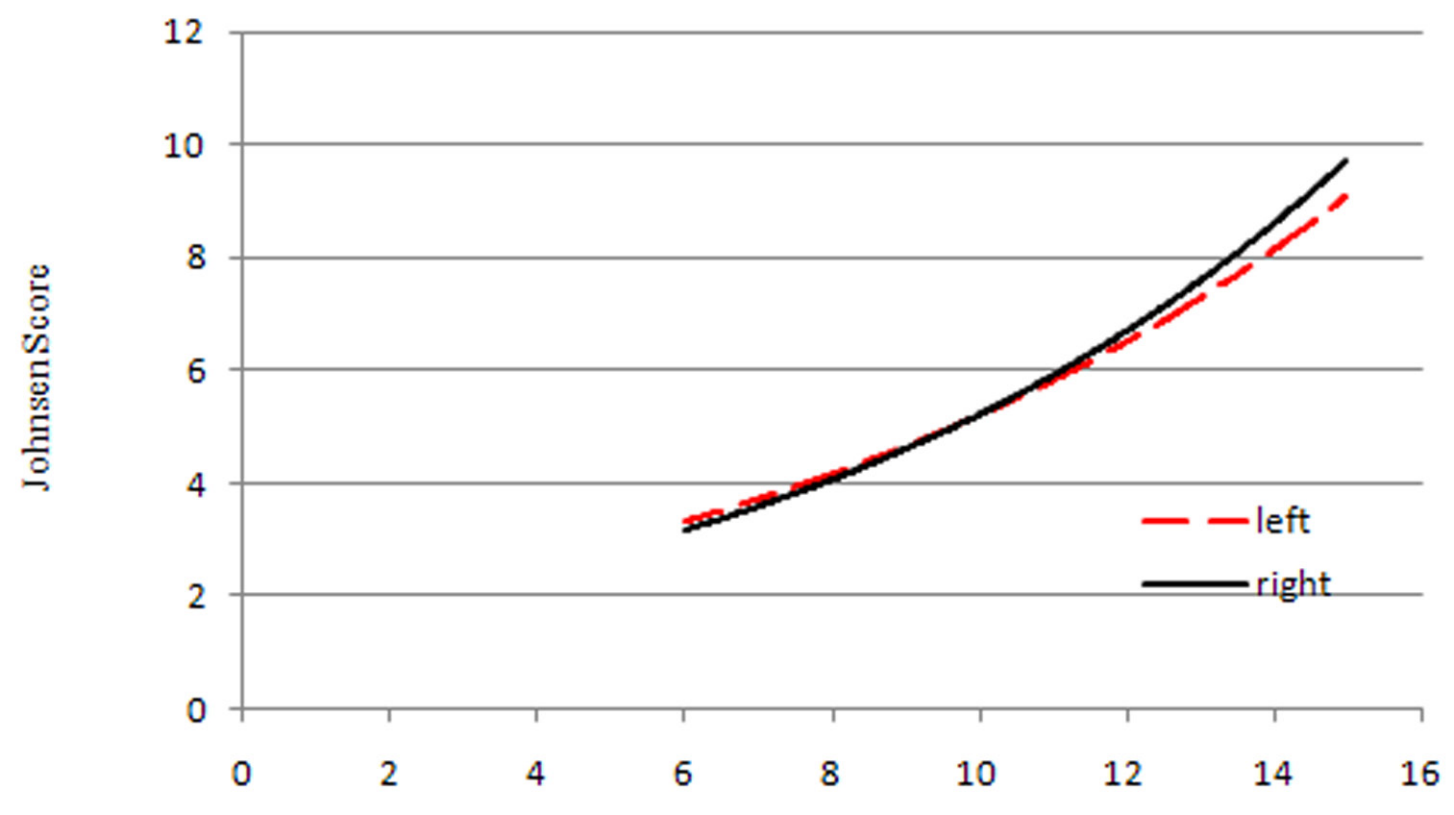
Trendlines between testicular volume and Johnsen score

The proportion of patients with a Johnsen score ≥8 for the left and right testes were 53.3% and 50.0%, respectively; Pearson’sχ^2^ test revealed these values were not statistically significantly different (*p*=0.266). The Cohen kappa value was 0.000 (*p*=1.000), revealing that the consistency of Johnsen score determination for the bilateral testes was caused entirely by random effects. Thus, the score for one side did not represent the other side (Table 2). According to testicular volume subgroup and analysis, we found there was a statistically significant difference between the left and right testes in the 10–12mLsubgroup or >12mLsubgroup; these differences were clearly related to differences in testicular volume (Table 2). Using left and right testicular volume, and corresponding Johnsen scores, to draw a trend-line, we revealed that Johnsen score increased with increasing testicular volume (Figure 3).

### Receiver operating characteristic (ROC) curve analysis

In our analysis, we set a testicular volume of 6 mL as the cut-off value and surgical indicator for percutaneous testicular puncture biopsy and puncture sperm retrieval. In order to increase the success rate of sperm retrieval and reduce unnecessary invasive procedures, we wanted to use ROC curves to optimize cut-off testicular volume and other parameters. In addition, we wanted to predict the accuracy and value of testicular sperm retrieval via evaluating testicular volume, along with serum PRL, FSH, LH, TT, E_2_ levels.

**Figure 4:**
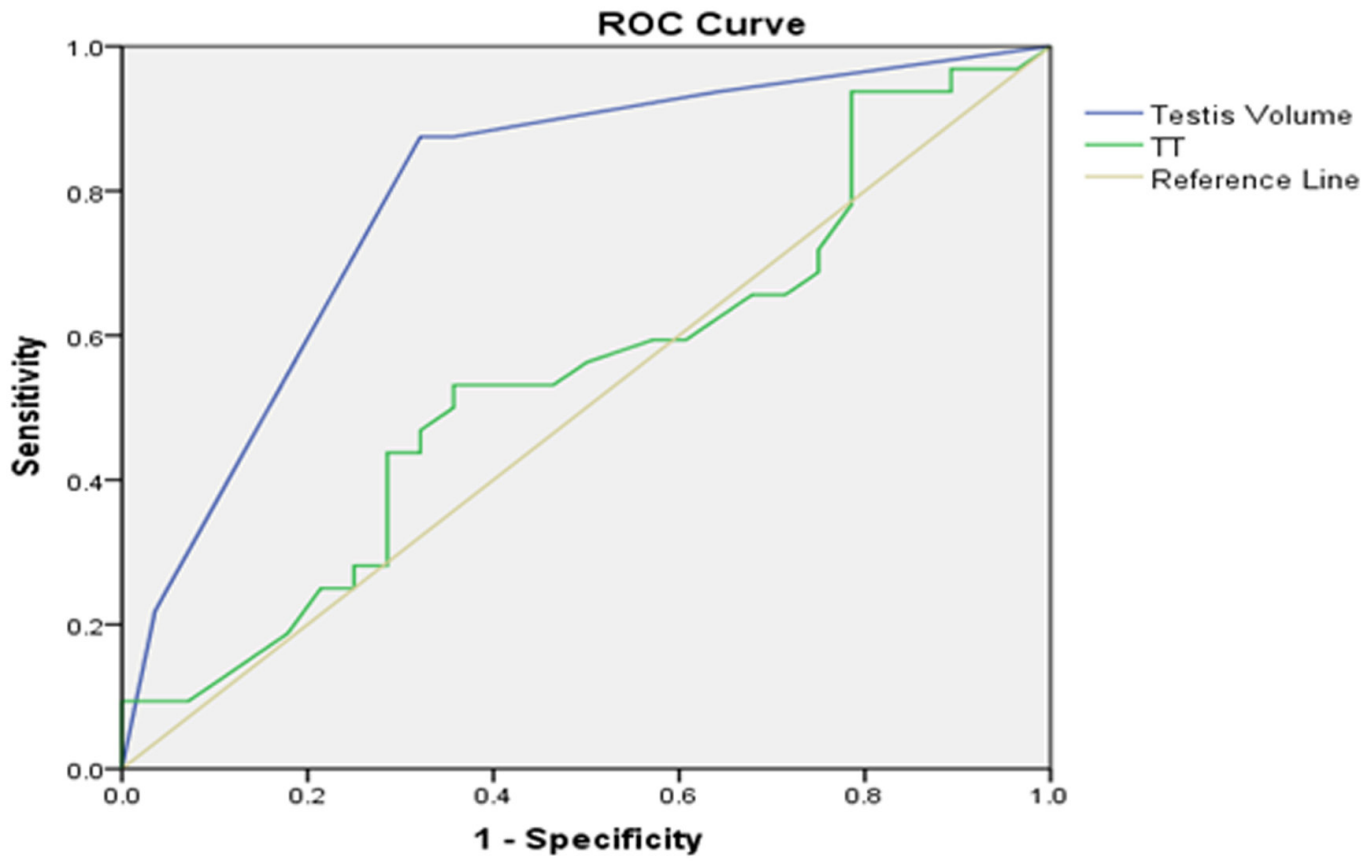
Left testicular Johnsen score≥8 as forecasted by receiver operating characteristic (ROC) curve analysis

**Figure 5:**
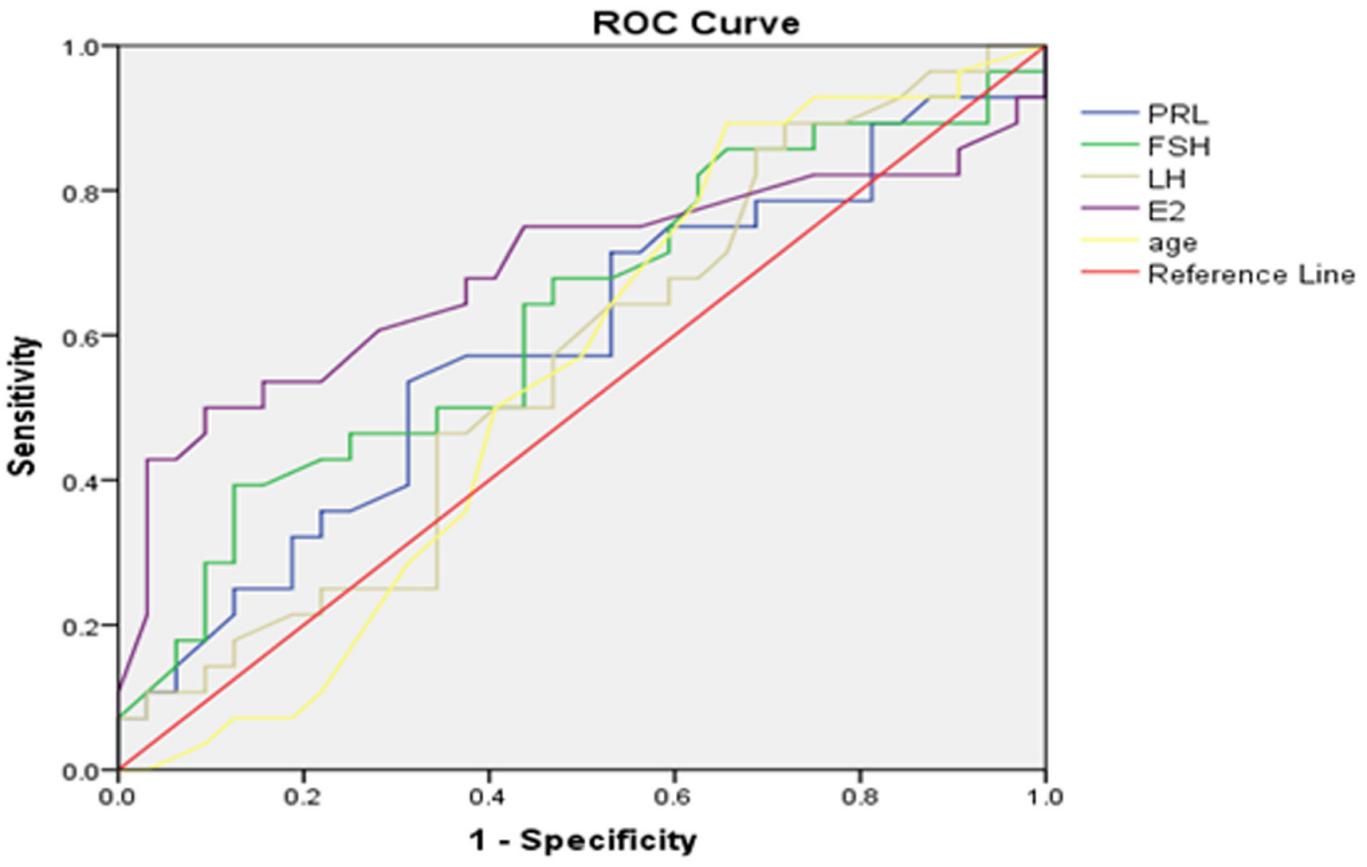
Left testicular Johnsen score≤7 as forecasted by receiver operating characteristic (ROC) curve analysis

**Figure 6:**
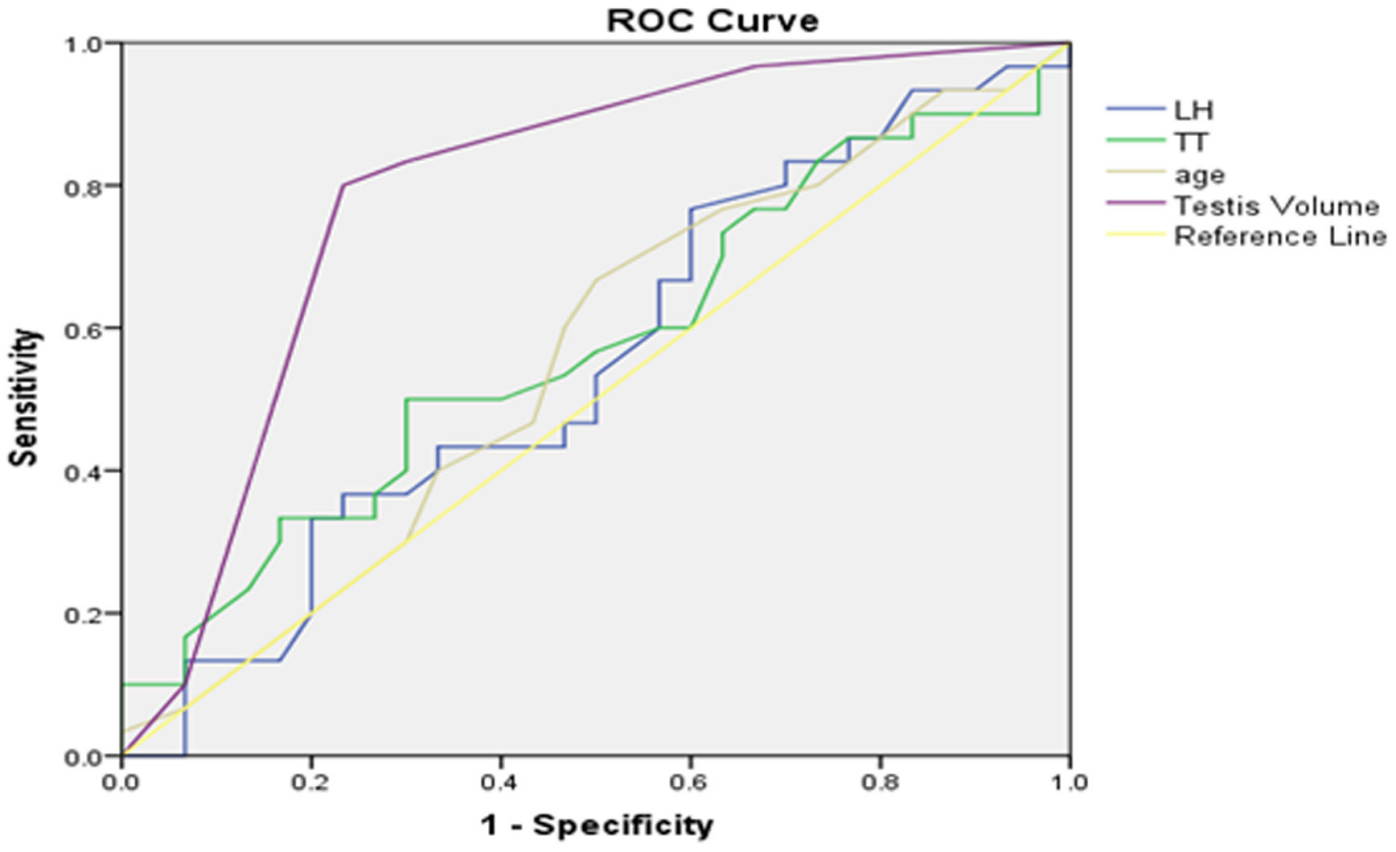
Right testicular Johnsen score≥8 as forecasted by receiver operating characteristic (ROC) curve analysis

**Figure 7:**
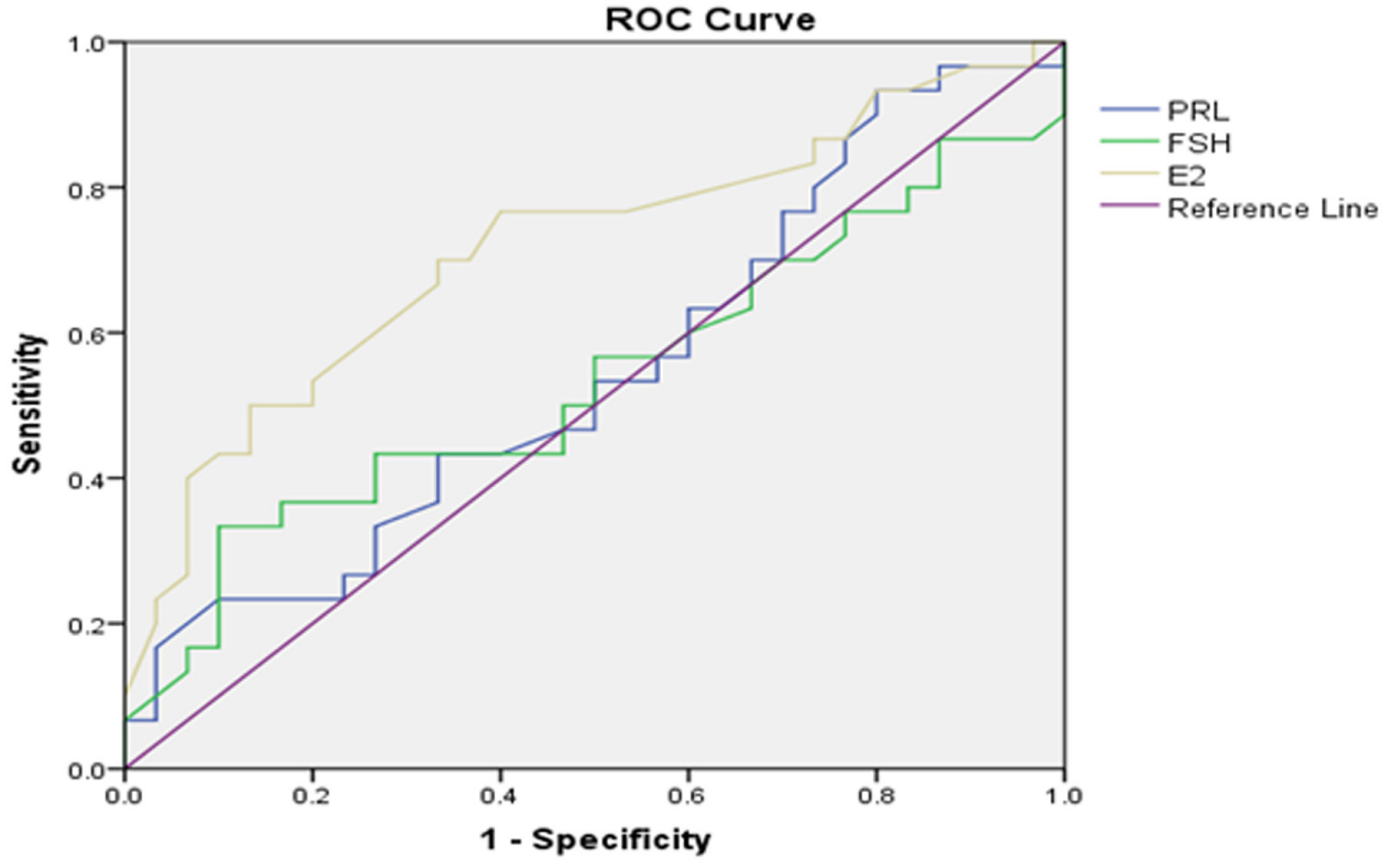
Right testicular Johnsen score≤7 as forecasted by receiver operating characteristic (ROC) curve analysis

Setting testicular histology Johnsen score as the gold standard to draw an ROC curve, we explored a range of parameters for their ability to forecast a Johnsen score ≥8. Area under the curve (AUC) for left testicular volume was0.796 (*p*=0.000,95%CI:0.729–0.863), while the AUC for right testicular volume was 0.791 (*p*=0.000,95%CI:0.721–0.860), optimized cut-off value for bilateral testicular volume was thus determined to be 11mL (Table 3). Furthermore, we explored the use of the same range of parameters to forecast a Johnsen score≤7. The AUC for E_2_ on left testis was 0.688 (*p*=0.000, 95%CI: 0.605–0.771), while the AUC for E_2_ on right testis was0.716 (*p*=0.000,95%CI: 0.641–0.792); optimized cut-off values of E_2_ on left and right testes were 144.5pmol/L and133.5 pmol/L, respectively. According to ROC curve and AUC analysis, we found that testicular volume and E2 represented significant indicators to estimate and forecast the presence or absence of testicular sperm in testicular biopsies, with moderate accuracy. However, age, serum PRL, FSH, LH and TT levels could not accurately predict the existence of testicular sperm. ROC curves are presented in Figures 4–7.

**Table 3:** AUC and statistics data of related parameters

	Johnsen Score	Parameters	Area Under the Curve	Std. Error	Asymptotic Sig.	Asymptomic 95% Confidence Interval	
						Lower Bound	Upper Bound
Left Testis	≥8	Testis Volume	0.796	0.034	0.000	0.729	0.863
		TT	0.544	0.043	0.308	0.459	0.629
	≤7	age	0.546	0.043	0.290	0.461	0.631
		PRL	0.587	0.043	0.044	0.503	0.671
		FSH	0.627	0.042	0.003	0.545	0.710
		LH	0.561	0.043	0.156	0.477	0.645
		E2	0.688	0.042	0.000	0.605	0.771
Right Testis	≥8	Testis Volume	0.791	0.035	0.000	0.721	0.860
		age	0.556	0.043	0.193	0.472	0.641
		LH	0.552	0.043	0.231	0.467	0.636
		TT	0.574	0.043	0.085	0.491	0.658
	≤7	PRL	0.547	0.043	0.274	0.463	0.632
		FSH	0.537	0.044	0.395	0.451	0.623
		E2	0.716	0.039	0.000	0.641	0.792

### Pearson correlations

Our analysis revealed significant correlation between age and finding sperm in the left testis (*p*=0.014) and with serum TT level (*p*=0.020). Correlations were also revealed between left testicular volume and finding sperm in the left testis (*p*=0.042), left testicular Johnsen score (*p*=0.000), serum FSH (*p*=0.000), LH (*p*=0.015), TT (*p*=0.047) and E2 (*p*=0.000) levels. There was also correlation between right testicular volume and right testicular Johnsen score (*p*=0.000), serum TT (*p*=0.000) and E2 (*p*=0.000) levels. Correlations were also detected between serum FSH level and finding sperm in the left testis (*p*=0.000), bilateral testicular Johnsen score (*p*=0.000) and serum TT level (*p*=0.017). Further analysis showed correlation between serum LH level and finding sperm in the right testis (*p*=0.000) and serum TT level (*p*=0.018), and between serum TT level and left testicular Johnsen score (*p*=0.019). Finally, correlation was revealed between serum E_2_ level and finding sperm in the left testis (*p*=0.001) and bilateral testicular Johnsen score (*p*=0.000).

## DISCUSSION

Our research found that the rates of positive sperm detection in the left and right testicular histopathological sections were 55.0% and 51.7% respectively, and that the proportions of patients with a Johnsen score ≥8 in the left and right testes were 53.3% and 50.0%, respectively. These data are similar to those reported in the existing literature (45%–63%; 2–5, 11–13). In general, when cTESE is used, the success rates of sperm retrieval in NOA patients are reported to be33.7%–50.9% [11, 12, 13, 14, 15, 16], however, lower rates are reported to be 16%–33% [17]. SRR from cTESE is lower than that for micro-TESE, which commonly shows rates of50.8%–56.0% [11, 12]. In a review of the literature, Esteves et al. revealed that TESA retrieval rates from NOA patients ranged from 10%–30%, except in cases of previously successful TESA or testicular histopathology showing hypospermatogenesis. In such cases, TESA SRR ranges from 70%–100% [17]. Another systematic review showed that the mean SRR for TESE was 49.5%, while another paper reported that the SRR for TESA and TESE was 41.8% and 50.9%, respectively (*p*= 0.04) [18]. The SRR calculated for our present study was higher than that reported previously for TESA data.

Over recent years, physicians have combined TESE with micro-TESE in order to improve SRR. Using the initial conventional biopsies, sperm could be retrieved in 46.8% of patients. With micro-TESE, sperm could be retrieved from an additional 11.4% of patients [11]. There was also a report showing that sperm could be detected in 33.7% of NOA patients using cTESE. Using micro-TESE, sperm detection increased to 50.8%, thus representing a statistically significance increase. Conventional TESE, combined with micro-TESE, can be used in selected patients with atrophic testes [12]. Another paper showed that testicular motile sperm were successfully retrieved in 82.9% of NOA men who had sperm detected in a previous biopsy (cTESE), and in15.3% of NOA men with no sperm in aprevious biopsy (*p*<0.01) [19].

In order to improve SRR for NOA patients, more and more physicians have adopted and used the therapeutic strategy of sequential or upgrade operations. The first type of strategy was repeated operations. Sperm were obtained in 44.5% of NOA patients after a single biopsy, but success rates increased to 51.4%,56.1%, 58.4%, and 59.5% after the second, third, fourth, and contralateral sampling, respectively. Multiple sampling thus increased the success rate; however, success rate did not increase considerably after the third sampling [20]. The second type of strategy was the use of different surgical techniques. Testicular sperm were found in 38.4% of NOA cases with TFNA as the first step, in 52.1% with TFNA and TESE, and in 64.4%with TFNA, TESE and micro-TESE. The three-step sperm retrieval method can thus significantly improve SRR for NOA patients. The third type of strategy was attempting micro-TESE for those patients in which physicians had previously failed to retrieve sperm. In those NOA men who had previously undergone unsuccessful procedures elsewhere, the SRR using micro-TESE was 57.1%. Micro-TESE should therefore be considered the gold standard for the retrieval of testicular sperm from NOA males, as a significant proportion of men undergoing micro-TESE would have successful sperm retrieval irrespective of previous histology or previous unsuccessful surgery [7]. The fourth type of strategy was improving SRR from AZFc microdeletion patients. NOA is caused in up to 10% of patients by microdeletions of the Y chromosome in the azoospermia factor (AZF) region. Successful sperm retrieval was possible only in patients with isolated AZFc microdeletions. The sperm detection rate for conventional multilobular TESE was 25%, and the sperm detection rate for micro-TESE was significantly higher at67%[21]. At present, the majority of physicians agree that micro-TESE yields the highest SRR, and that multiple TESE was superior to cTESE. Micro-TESE should therefore be preferred in severe NOA cases [22].

Research studies on the consistency of bilateral testicular histopathology and Johnsen score were previously insufficient. Our present results showed that although there was not statistically significant difference between left and right testicular histopathology in terms of sperm positive rates or Johnsen score, the consistency for Johnsen scoring for bilateral testes was caused entirely by random effects and that one side could not represent the other. Therefore, we recommend that the biopsy of both testes is required when NOA patients undergo biopsy for sperm retrieval. Existing literature supports this opinion. Performing contralateral testicular biopsy is advantageous in patients with uniform or mixed patterns of hypospermatogenesis. NOA patients who have undergone TESE should undergo at least three biopsies and additional biopsies might also be advantageous when the NOA was a consequence of either uniform or mixed pattern hypospermatogenesis [20]. Johnsen score remains an effective method with which to evaluate testicular histopathology in a quantitative manner and one of the independent predictive factors for detecting sperm [23]. Another study showed that the strongest predictor of the success of TESE was when tubules with mature spermatozoa (Johnsen score ≥8) were found in the histopathology specimen, irrespective of the overall state of spermatogenesis. (2) In general, Johnsen score was more significant for testicular biopsy and histology evaluation, and was helpful for forecasting the success rate of sperm retrieval.

On the basis of the above information, SRR of SCOS, maturation arrest and hypospermatogenesis were 27.6%–44.5%, 26.6%–66.7% and 75.86%–100% respectively. Thus, we believe that SRR is associated with the pathological nature of the testicular tissue, and is higher with hypospermatogenesis. However, some physicians have reported that histopathological findings did not associate with successful micro-TESE [24]. We used ROC curves and correlation analysis to show that testicular volume and serum E_2_ level correlated with Johnsen score, and that both of these parameters were more accurate predictors to estimate or forecast testicular sperm existence. Optimized cut-off value for bilateral testicular volume was 11mL, and optimized cut-off values for serum E_2_ levels in the left and right testes were 144.5 pmol/L and 133.5 pmol/L, respectively. Bonarriba et al. also reported cut-off points determined using ROC curves were 67 pg/mL for inhibin B (INH-B) and 12.2 mUI/mL for FSH [14].

We found that parameters to forecast testicular SRR have become the particular focus of research over recent years. Researchers have considered several parameters which could act as predictors, including age, testicular volume, histopathology (hypospermatogenesis and Johnsen score), serum FSH, INH-B, TT levels, Y chromosomal microdeletions and family history [8, 14, 24, 25]. However, other researchers have some that parameters could not be treated as predicators, including age, testicular volume, histopathology (hypospermatogenesis), previous negative TESE, serum FSH, LH, INH-B levels, seminal plasma INH-B, and anti-Müllerian hormone [7, 16, 21, 25, 26]. It was thus evident that there has been some disagreement between research studies with this respect.

Several papers have agreed that some of these positive predictors were very significant (Table 4). The first paper revealed that younger age was the only preoperative factor associated with successful sperm retrieval in NOA men with small testes (2 mLor less), although testicular volume did not affect SRR for micro-TESE. Men with the smallest volume testes, and those who were younger with Klinefelter syndrome, had the highest SRR. Severe testicular atrophy should not be a contraindication for micro-TESE. In addition, we should pay attention to family history or familial cases of NOA, because this factor influenced the success rate of testicular sperm retrieval. The family history of NOA patients should also be considered, including relatives with a history of infertility, deaths, abortions, or mental retardation in previous children. A positive history was an independent predictive factor for sperm retrieval. Sperm was most likely present in patients without a positive family history [23]. Another significant report involved patients divided into two groups; Group A with familial idiopathic NOA (11 families with two brothers in each family, 22 patients) and Group B with non-familial idiopathic NOA (97 patients). In Group A, the SRR for micro-TESE was 9.1% (2/22 patients) compared to 45.4% in Group B (44/97 patients) (*p* ≤ 0.05). The two patients in Group A with successful sperm retrieval belonged to one family. The histopathological diagnosis was the same in the brothers for each family [25]. The associated use of multiple parameters thus increased predicted value. A combination of serum FSH and testis size could thus substitute for invasive testis biopsy for predicting the existence of spermatozoa in infertile men with azoospermia; sensitivity for this technique was 77.3% and specificity was 85.2% [26]. Our results revealed that testicular volume and serum E_2_ level were accurate predictors with which to estimate or forecast the existence of testicular sperm, while age, serum PRL, FSH, LH, TT levels could not be treated as predictors; this finding concurred with some of the other previously published papers.

**Table 4:** Literatures on testicular histopathology pattern and sperm retrieval rates

Authors and publishing age (operation method)	Sperm retrieval rates of testicular histopathology (%)			
	SCOS (%)	Maturation arrest (%)	Hyposperma-togenesis (%)	Seminiferous tubules atrophy (%)
Robin G, et al.2010(TESE)(618)	16-33			
Kalsi J, et al.2012(mTESE)(607)	42.85	26.6	75.86	
Ma M, et al.2012(Three-Step Sperm Retrieval method)(622)	40.0	66.7	85.2	
Gul U, et al.2013(TESE)(626)	27.6			
Yildirim ME, et al.2014 (mTESE)(627)	35	40	100	31.03
Berookhim BM, et al. 2014 (mTESE)(628)	44.5			
Cetinkaya M, et al. 2015 (TESE)(625)	36	48.6	95.5	
Caroppo E, et al. 2017 (TESE)(609)	30.5	30.9	88.2	

We used correlation analysis to indicate there were correlations between testicular volume, serum FSH, E_2_ levels and testicular Johnsen score, but ROC curve analysis negated the predictive ability of FSH. Some previous literature has supported that there was a good correlation between the histology found upon diagnostic biopsy, testicular volume and the likelihood of finding mature sperm cells during testicular sperm retrieval or ICSI [12, 24]. However, other papers disagreed with this viewpoint [14, 24], and reported that there was no relationship between SRR and FSH levels [7, 12]. Researchers found that low INH-B and high FSH levels were correlated to sperm retrieval failure and that lower INH-B was more related to sperm retrieval failure than higher FSH. The lack of germ cells was also correlated with a high probability of sperm retrieval failure, while the presence of cryptozoospermia was correlated with a high probability of sperm retrieval success [14].

Our research was subject to some limitations. Firstly, we did not use statistical data for SRR of sequential or upgrade operations, for example, TESA or cTESE +micro-TESE. Secondly, we did not measure genetic parameters relating to spermatogenesis or NOA, although several genes have been reported in the literature. For example, *SOHLH1* (spermatogenesis and oogenesis-specific basic helix-loop- helix 1) represented an excellent candidate gene for testicular failure such as NOA, a splice-acceptor site mutation could create a non-functional *SOHLH1* protein, thus resulting in NOA due to the lack of normal spermatogenesis [27, 28]. The germline markers, stage-specific embryonic antigen (*SSEA-1*), *c-KIT* and *VASA* can be used as a complementary tool to create new molecular categories for diagnoses in azoospermic patients, which may be particularly useful to discriminate between mosaic and non-mosaic SCOS patients [29]. Assessing the expression of both *CDY1* and *BOULE* by qualitative reverse transcriptase-polymerase chain reaction (RT-PCR) has proved to be a sensitive and feasible test for predicting the presence of sperm cells in testicular tissue and may serve as a predictive tool if repeated TESE is required [30].*DAZ*, *AKAP4*, *PRM1*, and *PRM2*are testis stage-specific genes. The presence of *DAZ* and *PRM2* transcripts in semen was a significant indicator for the presence of spermatogonia and spermatids in testicular tissues while the absence of all four markers in semen confirmed the histopathological results corresponding to SCOS. Although TESE should not be excluded solely upon this criterion, using *PRM1*, *PRM2*, *AKAP4*, and *DAZ* transcripts as markers would provide an excellent non-invasive molecular diagnostic tool to better counsel patients before undergoing TESE [31]. However, we have still to devise a set of perfect parameters with which to accurately forecast the existence of testicular sperm and apply such parameters in normal clinical practice.

## MATERIALS AND METHODS

### Patients

This was a retrospective research study involving180 consecutive patients with NOA who underwent testicular biopsy by TESA to obtain testicular tissue and to evaluate Johnsen score of bilateral testicular histopathology between May 2016 and November 2016. Patients attended the infertility clinics of two hospitals attached to Peking University. Azoospermia was confirmed by the analysis of at least two different centrifuged (3000×*g*, 15min) semen samples in accordance with World Health Organization (WHO) criteria. Testicular volume was measured by physical examination with a Prader or chidometer to confirm that at least one testis had a volume ≥6mL. Serum endocrine profile, such as follicle stimulating hormone (FSH), luteinizing hormone (LH), total testosterone (TT), estrodial (E_2_) and prolactin (PRL) was ascertained for each patient along with karyotyping and Y chromosomal microdeletion analysis. Patients with obstructive azoospermia, hypogonadotropic hypogonadism, karyotype abnormality and AZFa or AZFb microdeletions were excluded from our analysis. Karyotyping for three of the patients showed 46, XY, 1qh+, 46, XY, 13psk+, 46, XY, Yqh+ and 1 of the patients showed AZFc microdeletion; all other patients were normal. All patients had been diagnosed with NOA and required testicular biopsy and histopathology to evaluate the status of spermatogenesis and testicular sperm in order to provide patients with sufficient knowledge to make a judgement as to whether or not to proceed to ICSI. All patients provided informed written consent. In our hospitals, a bilateral testicular volume of <6mL in an NOA patient is an indicator for micro-TESE. Consequently, any such patients did not have any histopathology results and were not enrolled into our study.

### Surgical technique of testicular biopsy (TESA)

We used spermatic cord block and local anesthesia. An18G-needle attached to a syringe was percutaneously inserted into one side of the testis. A piece of testicular tissue was punctured and aspirated out of each testis, fixed in Bouin’s fluid and sent for histopathology examination in the pathology department.

### Histopathology examination

Testicular tissues were cut into pathological sections and stained using a routine hematoxylin–eosin staining method (HE). Sections were then diagnosed by specially-assigned doctors who evaluated pathological findings, determined Johnsen score and assessed for the presence or absence of sperm.

### Serum endocrine profiling

Blood was drawn from the veins of all180 patientsbetween07:30 and 10:00 h. Routine chemiluminescence assays were then performed to determine serum levels of FSH, LH, TT, E2 and PRL.

### Statistical analysis

Microsoft Excel 2007 software (Microsoft Corporation, 15700 NE 39th St, Redmond, WA 98052) and SPSS21.0 (IBM Corporation, 1 New Orchard Road, Armonk, New York 10504-1722, United States) were used for all statistical analysis. Tests were considered statistically significant if *p*<0.05.

## CONCLUSIONS

While there were no statistically significant differences between left and right testicular histopathology in terms of sperm positive rates or Johnsen score, the consistency of Johnsen scoring for bilateral testes was caused entirely by random effects and the score for one side could not represent the other side. Therefore, we recommend that both testes require surgery when NOA patients undergo biopsy or sperm retrieval.
